# TIMI perfusion grade compared to TIMI flow in prediction of infarct size and microvascular obstruction measured by contrast-enhanced MRI

**DOI:** 10.1186/1532-429X-11-S1-P231

**Published:** 2009-01-28

**Authors:** Georg FK Fuernau, Kathrin Schindler, Ingo Eitel, Josef Friedenberger, Eigk Grebe, Gerhard Schuler, Holger Thiele

**Affiliations:** University of Leipzig – Heartcenter, Leipzig, Germany

**Keywords:** Myocardial Infarction, Infarct Size, TIMI Flow, Left Ventricular Mass, Prognostic Impact

## Background

The TIMI perfusion grade (TMPG) and TIMI flow are angiographic parameters with prognostic impact in ST-elevation myocardial infarction (STEMI). The association of these parameters with infarct size and microvascular obstruction assessed by MRI, which also have of prognostic impact, has not been studied so far.

## Methods

From 02/2006–01/2008 280 consecutive patients with STEMI underwent primary PCI. In an MRI study (day 1–4 after PCI) infarct size and microvascular obstruction in percent of left ventricular mass (%LV) were measured by delayed enhancement. In addition, post-PCI TIMI flow and TMPG were assessed from invasive angiography. All measurements were done by blinded investigators. TMPG and TIMI flow were graded as severely impaired (0–1) or mildly impaired-normal (2–3).

## Results

For post-PCI TIMI flow there was a significant difference in the extent of microvascular obstruction (TIMI 0–1: 6.6 ± 5.1%LV vs. TIMI 2–3: 3.6 ± 4.6%LV, p = 0.021), but no significant difference in infarct size (TIMI 0–1: 25.9 ± 15.3%LV vs. TIMI 2–3: 21.19.3 ± 13.4, p = 0.07). For TMPG microvascular obstruction (TMPG 0–1: 5.3 ± 5.2%LV vs. TMPG 2–3: 3.5 ± 4.5%LV, p = 0.011) and infarct size were significantly different (TMPG 0–1: 25.0 ± 13.0%LV vs. 18.6 ± 13.5%LV, p = 0.002). In a multivariable model the strongest predictors of infarct size and microvascular obstruction were post-PCI TMPG, infarct location, Killip class, and 90 minute ST-segment resolution (p < 0.005 for all).

## Conclusion

In STEMI patients undergoing primary PCI the TMPG is a better indicator of angiographic success compared to TIMI flow reflected by a significant difference in infarct size measured by cardiac MRI. This might explain why the TMPG has additional prognostic impact in patients with restoration of normal TIMI flow.

